# Neural responses to sounds presented on and off the beat of ecologically valid music

**DOI:** 10.3389/fnsys.2013.00014

**Published:** 2013-05-10

**Authors:** Adam Tierney, Nina Kraus

**Affiliations:** ^1^Department of Communication Sciences, Auditory Neuroscience Laboratory, Northwestern UniversityEvanston, IL, USA; ^2^Department of Communication Sciences, Northwestern UniversityEvanston, IL, USA; ^3^Institute for Neuroscience, Northwestern UniversityEvanston, IL, USA; ^4^Department of Neurobiology and Physiology, Northwestern UniversityEvanston, IL, USA; ^5^Department of Otolaryngology, Northwestern UniversityEvanston, IL, USA

**Keywords:** auditory, brainstem, cortex, music, rhythm

## Abstract

The tracking of rhythmic structure is a vital component of speech and music perception. It is known that sequences of identical sounds can give rise to the percept of alternating strong and weak sounds, and that this percept is linked to enhanced cortical and oscillatory responses. The neural correlates of the perception of rhythm elicited by ecologically valid, complex stimuli, however, remain unexplored. Here we report the effects of a stimulus' alignment with the beat on the brain's processing of sound. Human subjects listened to short popular music pieces while simultaneously hearing a target sound. Cortical and brainstem electrophysiological onset responses to the sound were enhanced when it was presented on the beat of the music, as opposed to shifted away from it. Moreover, the size of the effect of alignment with the beat on the cortical response correlated strongly with the ability to tap to a beat, suggesting that the ability to synchronize to the beat of simple isochronous stimuli and the ability to track the beat of complex, ecologically valid stimuli may rely on overlapping neural resources. These results suggest that the perception of musical rhythm may have robust effects on processing throughout the auditory system.

## Introduction

One of the fundamental characteristics underlying speech and music is organization in time. In music, alternating strong and weak segments form a metrical rhythm with an underlying steady beat, one of the only characteristics of music that is widely present across cultures (Nettl, 2000). In speech, stressed and unstressed syllables occur in somewhat predictable temporal patterns. Speech rhythm is resistant to acoustic degradation, making it a useful cue for word segmentation in noise (Smith et al., 1989).

It has been proposed that both speech rhythm (Goswami, 2011) and musical rhythm (Large, 2008) are tracked via entrainment of neuronal oscillators. Phase-locking of neuronal oscillations to slow rhythms in speech and music may lead to alternating periods of greater and lesser salience (Large and Jones, 1999), as the phase of neural oscillations within the 2–6 Hz range has been linked to acoustic target detection (Ng et al., 2012) and both auditory and visual perceptual processing are enhanced when stimuli are aligned with musical beats (Jones et al., 2002; Escoffier et al., 2010). Stimuli presented at times aligned with a perceived musical beat may, therefore, lead to greater firing rates and evoked electrophysiological potentials. If shared neural processes do underlie rhythm tracking in music and speech, delineating these processes could lead to insights into normal and impaired functioning in both domains. For example, difficulty tracking slow temporal patterns has been suggested as a potential cause underlying language impairment (Abrams et al., 2009; Goswami, 2011).

Music is acoustically complex. Research on the neural correlates of metrical rhythm processing has, therefore, focused on the perception of trains of isochronous, identical sounds. Listeners tend to hear these as alternating strong and weak sounds, and this perceived metrical structure changes the brain's response. Perceived “strong” beats, for example, elicit enhanced responses to deviances (Brochard et al., 2003; Abecasis et al., 2005; Pablos Martin et al., 2007; Geiser et al., 2009, 2010; Ladinig et al., 2009; Potter et al., 2009; Winkler et al., 2009), oscillatory responses (Snyder and Large, 2005; Iversen et al., 2009), and N1 and P2 potentials (Abecasis et al., 2009; Schaefer et al., 2011; Vlek et al., 2011). It is unknown, however, whether these findings generalize to the perception of complex, ecologically valid music. It is also unknown whether metrical rhythm perception affects auditory brainstem function.

Our goal was to examine the effects of alignment with the beat of ecologically valid music on the neural encoding of sound. We hypothesized that phase-locking of slow neural oscillations to the beat of music decreases the threshold for firing and facilitates neural synchrony at beat onsets. To test this hypothesis we repeatedly presented subjects with a target sound embedded in background music. The target sound was either aligned with the musical beat or shifted later in time. Electrophysiological data were recorded from a single active electrode at a high sampling rate, allowing us to analyze both cortical and brainstem responses by selectively filtering and reanalyzing a single recording.

This paradigm enabled us to ask three novel questions. First, is the cortical processing of complex, ecologically valid musical rhythm qualitatively different from the processing of metrical rhythm reported by previous studies using simpler stimuli? Second, can rhythmic context modulate the encoding of sound within the auditory brainstem? And third, how does the effect of musical beat perception on auditory neural processing relate to the ability to synchronize movements to a beat?

## Material and methods

### Subjects

Thirty young adults (ages = 18–38, mean = 24.2, *SD* = 4.8), 20 female, participated in this study. Participants included subjects with a wide range of musical experience (0–29 years, mean = 8.7, *SD* = 8.2). All participants had pure tone air conduction thresholds ≤20 dB HL from 0.125–8 kHz and normal brainstem responses to a click (ABR wave V latencies within the normal range of 5.414–5.967) as measured using a Bio-logic Navigator system (Natus Medical). Informed consent was obtained in accordance with Northwestern University's Institutional Review Board.

### Stimulus presentation

Our electrophysiological paradigm was adapted from a tapping test developed by Iversen and Patel (2008). The target stimulus was a 200 ms synthesized bassoon tone with a 100 Hz pitch. The sound was presented at a +11 dB signal-to-noise ratio over the background music, which consisted of three pieces: “Keep the Customer Satisfied” by Paul Simon (duration: 158 s), “Jingles” by James P. Johnson (206 s), and “Pills” by Bo Diddley (171 s). These three pieces were chosen after an extensive search through recordings because each conveys a strong rhythmic feeling but lacks large amplitude differences between on-beat and off-beat times. Furthermore, each background music stimulus was hard-limited by 15 dB to eliminate amplitude spikes. For each of these pieces, the onset time of each musical beat was determined by having a professional drummer tap to the song on a NanoPad2 (Korg) tapping pad. The resulting mean intervals between beats in the three songs were 465, 443, and 416 ms. Because real, ecologically valid music was used, the music contains slight variations in tempo, and as a result there is no way to objectively assess the accuracy with which the drummer was able to reproduce the rhythms of the song. Nevertheless, the drummer produced a very consistent beat: the standard deviation of inter-tap intervals for the three songs was 14.29, 17.48, and 13.89 ms. In every case, therefore, the variation of the beat produced by the drummer was below the conscious threshold for detection of perturbations in a metronomic beat (20 ms; see Madison and Merker, 2004).

During the on-the-beat condition, the bassoon sound was presented such that its onset coincided with the time of each musical beat. The off-the-beat condition was identical to the on-the-beat condition, except that the target stimulus onset times were shifted later by one-fourth of the average interval between every musical beat in a given song; effectively, the stimuli were “out of phase” with the beat. Each song was presented twice during each condition, resulting in over 2000 stimulus presentations per condition.

The bassoon stimulus sequences in the two conditions were identical: the only difference between the conditions was in the relationship between the stimulus and the music. Nevertheless, if the amplitude of the background music were greater during the on-the-beat presentation times, a difference in simultaneous masking between the two conditions could potentially affect the neural response to the stimulus. To ensure that background music amplitudes did not differ between the two conditions, for each musical piece the average amplitude of the music during the 200 ms following each beat onset was calculated. *T*-tests revealed that, for all three pieces, amplitudes of the background music during stimulus presentation did not significantly differ between the two conditions (all *p* > 0.05). Moreover, the on-the-beat portions of the background stimuli had a mean amplitude of 0.0752, while the off-the-beat portions of the background stimuli had a mean amplitude of 0.0758, for a difference of approximately 0.07 dB, a difference that falls far below the threshold for psychophysical amplitude change detection of roughly 2 dB (Jesteadt et al., 1976). Similarly, for all three pieces the average amplitude of the background music during the 25 ms following beat onsets (mean amplitude = 0.0751) did not differ from the average amplitude during the 25 ms following shifted onsets (mean amplitude = 0.0752, *p* > 0.05), confirming that musical beats were not marked by sudden increases in amplitude.

To rule out frequency-specific masking, we used fast Fourier transforms (FFTs) to measure the frequency spectrum of the background music during the 200 ms following beat onsets and shifted onsets, then averaged amplitudes at 100, 200, 300, 400, and 500 Hz (corresponding to the fundamental frequency and first four harmonics of the bassoon stimulus). For each of the three pieces, FFT amplitudes did not differ between the two conditions at these five frequencies, confirming that frequency-specific masking was not more present in either condition (*t*-tests, all *p* > 0.05). Finally, to further ensure that simultaneous masking was not driving our results, we ran cross-correlations between the brainstem responses to the target sound in the two conditions. If masking were greater in either condition, the response to the on-the-beat tones would have occurred later (Burkard and Hecox, 1983). The lag at which the correlation was maximized did not significantly differ from zero (*t* = −1.16, *p* = 0.26), confirming the lack of simultaneous masking.

### Electrophysiological recording

Electrophysiological data were collected from Cz using Scan 4.3 Acquire (Compumedics, Charlotte, NC) with Ag-AgCl scalp electrodes at a sampling rate of 20 kHz and with open filters (0.1–3000 Hz). Electrodes were applied in a vertical, linked-earlobe-reference montage. The use of a linked earlobe reference can lead to a potential shifting of the effective reference (Miller et al., 1991), a factor which could be introducing ambiguity into our data. However, this effect is unlikely to be contributing to the difference between conditions we find, as both conditions were conducted in a single recording session and therefore any effects of the linked reference should be similar across the two conditions. These recording parameters are optimal for the collection of auditory brainstem responses (Galbraith et al., 1995; Chandrasekaran and Kraus, 2010). Contact impedance was 5 kΩ or less across all electrodes. This procedure enables brainstem and cortical responses to be recorded simultaneously. Stimuli were presented binaurally via insert earphones at 70 dB (ER-3; Etymotic Research, Elk Grove Village, IL). Target stimuli were presented in alternating polarities (i.e. every other stimulus was multiplied by −1) to minimize the contamination of the brainstem response with stimulus artifact and cochlear microphonic. During recording, participants were told to ignore the stimuli and watch subtitled, muted videos of their choice to ensure that they remained alert.

### Behavioral tests

#### Synchronized tapping

Subjects were tested on a synchronized tapping task adapted from Thomson et al. (2006). A snare drum sound was isochronously presented over speakers to subjects, who were asked to tap along to the beat on a NanoPad2 (Korg) tapping pad. Each trial began with the presentation of 20 practice beats, to give subjects ample time to synchronize to the beat. In a “paced” condition, after the conclusion of the practice section the beat continued without pause for another 20 beats while tapping times were recorded. In an “unpaced” condition, after 20 practice beats the sound presentation ceased and the subject was asked to continue tapping at the rate at which the metronome had been beating. Metronome beats were presented at 1.5, 2, and 3 Hz. Subjects' synchronization ability was scored based on the variability of their tapping responses, as calculated by computing the standard deviation of the intervals between taps. A composite score was created for both paced and unpaced conditions by averaging variability at all three tapping rates.

### Data analysis

Electrophysiological response averages were created offline. Responses were segmented into epochs spanning 50 ms before and 250 ms after each stimulus onset. Epochs from the three background songs were combined to ensure that any effect of rhythmic context found was not due to acoustic characteristics specific to a particular recording. To isolate the contribution of the cortex, responses were bandpass filtered from 0.1 to 20 Hz (12 dB/octave roll-off.) Responses were baselined to the pre-stimulus period of the response. Responses with activity ≥ ±75 μV were rejected as artifacts, and 2000 remaining sweeps per condition were averaged. The amplitude of the cortical onset wave P1 was calculated as the mean amplitude within 60 ms of the time of occurrence of the P1 peak in the inter-subject average (50–110 ms). The amplitude of the cortical wave N1 was also calculated as the mean amplitude within 60 ms of the time of occurrence of the N1 peak in the inter-subject average (205–265 ms). P1 and N1 amplitudes in the On-the-beat and Off-the-beat conditions were compared using paired *t*-tests. Grand averages were created for the two conditions by averaging across all 30 subjects.

To isolate the contribution of the brainstem, responses were bandpass filtered from 70 to 2000 Hz (12 dB/octave roll-off). Responses were baselined to the pre-stimulus period of the response. Responses with activity ≥ ±35 μV were rejected as artifacts, and 2000 remaining sweeps per condition were averaged. The amplitude of the brainstem wave V was calculated as the maximum amplitude within 1 ms of the time of occurrence of the wave V peak in the inter-subject average (12.75–13.75 ms). Wave V amplitudes in the On-the-beat and Off-the-beat conditions were then compared using a paired *t*-test. To examine the brainstem's encoding of the frequency content of the target sound, FFTs were taken of the frequency-following portion of the brainstem response (20–200 ms). Amplitudes in 10-Hz windows around the fundamental frequency (100 Hz) and the second through eighth harmonics averaged together were compared with paired *t*-tests. Correction for multiple comparisons was performed using a Bonferroni correction.

To determine the behavioral relevance of the neural encoding of the beat, the neural effects of beat perception were correlated with synchronized tapping performance.

## Results

### Electrophysiological effects of musical rhythm

**Figure 1:**
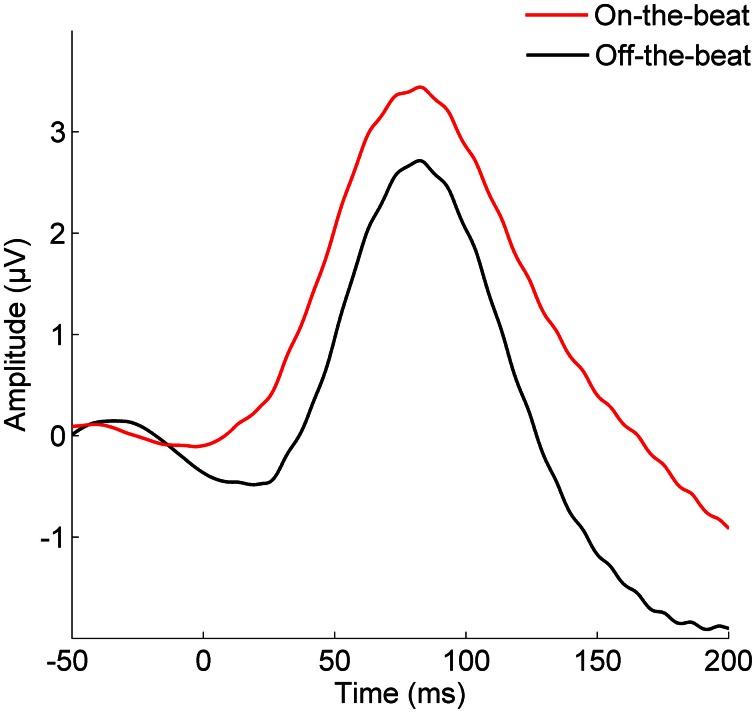
**The effect of musical rhythm on cortical sound processing.** The cortical P1 onset response is enhanced when a target sound is aligned with the beat of the music, rather than shifted away from the beat (paired *t*-test, *t* = 4.79, *p* < 0.0001).

**Figure 2:**
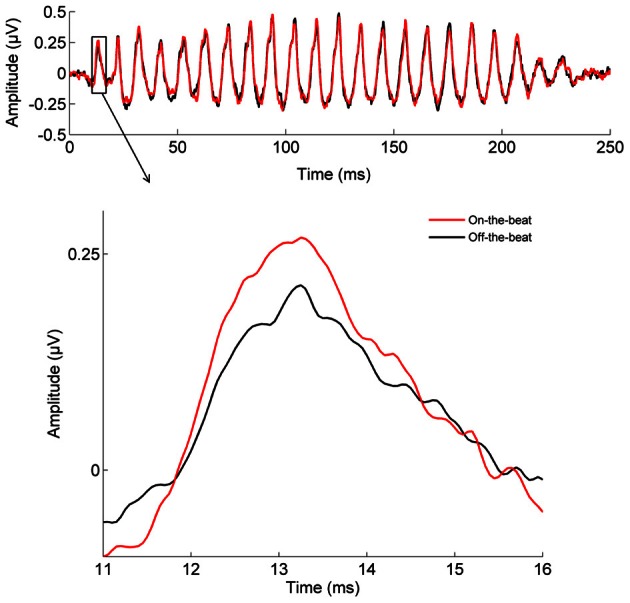
**The effect of musical rhythm on subcortical sound processing.** The brainstem wave V onset response is enhanced when a target sound is aligned with the beat of the music, rather than shifted away from the beat (paired *t*-test, *t* = 2.86, *p* < 0.01).

The cortical P1 response (Figure 1) was larger in the On-the-beat (2.99 μV) condition than the Off-the-beat (2.14 μV) condition (paired *t*-test, *t* = 4.79, *p* < 0.0001), confirming that alignment with the musical beat enhanced the cortical onset response to sound. The cortical N1 response, on the other hand, was smaller in the On-the-beat (−1.16 μV) condition than the Off-the-beat (−1.83 μV) condition (paired *t*-test, *t* = 2.99, *p* < 0.01). The brainstem wave V (Figure 2) was also larger in the On-the-beat (0.350 μV) condition than the Off-the-beat (0.289 μV) condition (paired *t*-test, *t* = 2.86, *p* < 0.01), confirming that alignment with a musical beat enhanced the brainstem onset response to sound. The effect was specific to wave V, as brainstem frequency encoding did not differ between the two conditions (paired *t*-tests; for the fundamental frequency, *t* = 0.26, *p* > 0.1; for the harmonics, *t* = 0.74, *p* > 0.1). The size of the cortical and brainstem effects (i.e., the difference between onset magnitudes in the two conditions) did not correlate (*r* = 0.0079, *p* > 0.1), suggesting that brainstem and cortical effects reflect two independent aspects of rhythm processing.

### Behavioral relevance

**Figure 3:**
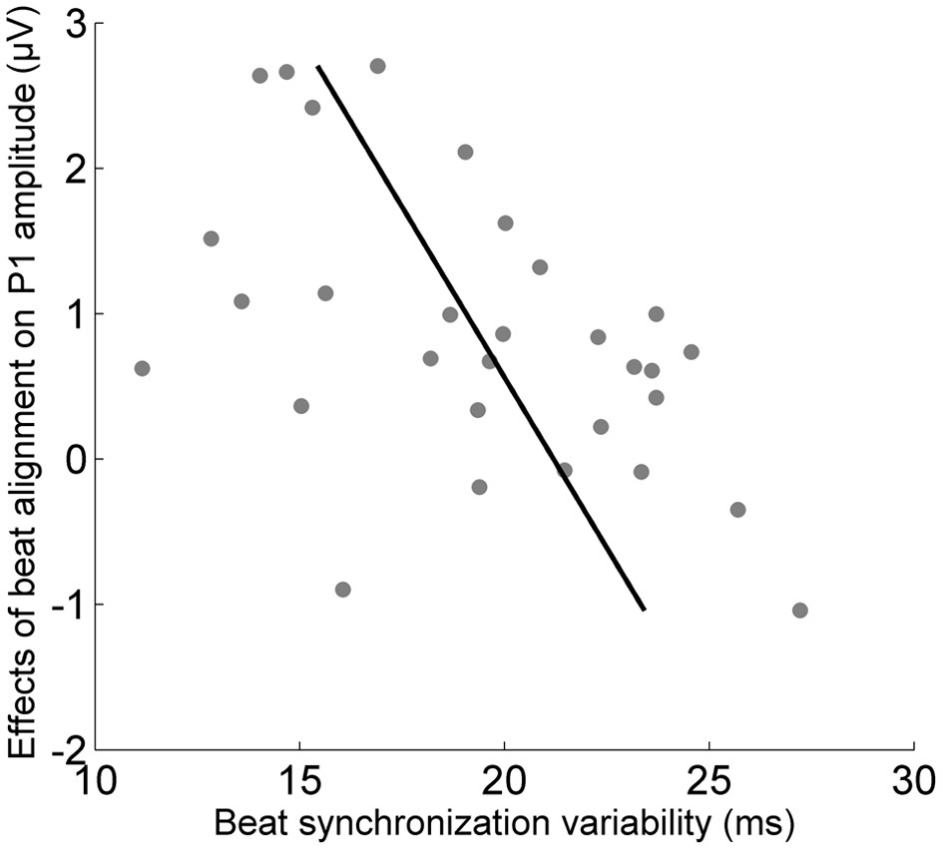
**Relationship between the cortex's sensitivity to the musical beat and beat synchronization ability.** The effect of the musical beat on the cortical response to sound relates to beat synchronization ability (*p* < 0.01).

To determine the behavioral relevance of the effects of alignment with the beat on cortical and brainstem onset responses, relationships between the size of the cortical and brainstem effects and synchronized tapping ability were measured using Pearson's correlations (Figure 3). The magnitude of the effect of beat alignment on the cortical onset significantly correlated with the synchronized tapping variability composite measure for the paced condition (*r* = −0.50, *p* < 0.05) but not the unpaced condition (*r* = −0.33, *p* > 0.1). Tapping ability in paced and unpaced conditions did not correlate with the size of the brainstem effect (for paced, *r* = 0.10, *p* > 0.1; for unpaced, *r* = 0.27, *p* > 0.1).

### Effects of musical experience

To determine whether a high degree of musical experience was necessary for subjects to show a difference between the two conditions, two repeated-measures ANCOVAs were conducted on the brainstem and cortical onset data. In both, condition (on- vs. off-the-beat) was the within-subjects factor while total number of years of musical experience was included as a covariate. For the cortical onset response there was an effect of condition [*F*_(1, 28)_ = 5.266, *p* < 0.05]. For the brainstem onset response there was a trending effect of condition [*F*_(1, 28)_ = 4.093, *p* < 0.1].

## Discussion

We examined the effects of musical rhythm on the brain's processing of sound by presenting a target sound either aligned with, or shifted away from, the beat of simultaneously presented music. We found that onset responses to the target sound in both the cortex (the P1 response) and the brainstem (wave V) were enhanced by alignment with the musical beat. This enhancement may stem from phase-locking of ongoing oscillations within auditory cortex and the brainstem to the beat of the music (Large, 2008), decreasing the threshold for firing at beat onsets.

Our findings are qualitatively different from the previous results reported in the electrophysiology literature on metrical rhythm processing: no previous study has demonstrated an effect of metrical rhythm on P1 amplitude. This result suggests that the effects of musical rhythm on the auditory system may be more immediate and more pervasive than has been previously supposed. As P1 is generated within primary and secondary auditory cortex (Godey et al., 2001), this finding suggests a specific locus for effects of rhythmic context on sound processing within the cortex. The discrepancy between our findings and those previously reported could be due to the fact that previous work used simple sequences of tones or clicks to elicit a rhythmic percept, while we used actual pieces of popular music. These findings are in line with prior work (Bolger et al., 2013) demonstrating that the elicitation of a rhythmic percept using ecologically valid stimuli can lead to greater behavioral effects than when abstract stimuli are used. Our study, therefore, suggests that the use of ecologically valid stimuli may be fruitful when studying complex auditory signals such as speech or music.

The N1component was smaller when the stimulus was aligned with the beat, compared to when it was shifted away from the beat. This effect is consistent with the findings of Cason and Schön (2012), who also showed an N1 enhancement for stimuli presented at off-beat versus on-beat times. The N1 is generated by at least three different components (Näätänen and Picton, 1987) likely stemming from auditory and frontal cortex (Giard et al., 1994). The physiological mechanism underlying its off-the-beat enhancement is, therefore, difficult to determine from these data. Nevertheless, this effect lends support to the idea that the N1 can be enhanced by a violation of rhythmic expectations (Cason and Schön, 2012).

Prior work has established that the brainstem is sensitive to a sound's surrounding context, and that the extent of this context-sensitivity is linked to reading ability and the ability to perceive speech in noise (Chandrasekaran et al., 2009; Parbery-Clark et al., 2011; Strait et al., 2011). We find, for the first time, that the onset component of the auditory brainstem response to sound can be modulated by the rhythmic context in which the sound is embedded. Specifically, Wave V was enhanced; this wave is generated by the lateral lemniscus and inferior colliculus (Hood, 1998), suggesting that the modulatory effects of rhythmic context on sound processing extend to these regions of the brainstem. The mechanisms underlying the brainstem's sensitivity to rhythmic context remain to be delineated. One clue, however, is provided by the fact that rhythm's effect on the cortical onset response did not correlate with its effect on the brainstem onset response. This suggests that the effect of musical rhythm on auditory processing within the brainstem and the cortex stems neither from strict bottom-up propagation nor top-down modulation. Instead, tracking of rhythmic patterns and the resulting modulation of auditory processing may take place locally in parallel in both the cortex and the brainstem.

The ideal stimuli to use to study musical rhythm perception would be ecologically valid pieces of music that are nonetheless perfectly controlled in every respect, such that no one part of the music differs from any other in any musical or acoustic attribute. Unfortunately, any stimulus that was thus constructed would then fall outside the bounds of ecologically valid music. Instead, we found/constructed stimuli that were controlled for basic acoustic attributes such as waveform amplitude and frequency spectrum. However, given our goal of examining the effects of perception of ecologically valid music, we could not perfectly control for musical characteristics of the background such as harmony, contour, and consonance. It is possible, therefore, that differences in the background music in these respects are driving our results. Specifically, rapid changes in harmony, contour, or consonance, among other musical characteristics could occur around beat onset times, potentially modulating the neural response to the concurrently presented stimulus. Although we suggest, therefore, that the cortical P1 and subcortical wave V modulation we find may due to the rhythmic percept, future work should substantiate this finding by attempting to eliminate confounding factors. It may be possible, for example, to construct stimuli that occupy a middle ground between the simple, abstract stimuli often used when studying rhythm and stimuli with the richness and complexity of real music. The ideal stimuli—which may or may not be possible to construct—would be simple enough that they could be controlled for all acoustic and musical characteristics, but ecologically valid enough to generate a rhythmic percept nearly as strong as that found in real music.

Although the effect of alignment with the beat on the cortical onset response was strong, there was nonetheless substantial variation in the size of the effect among subjects. Individual differences in the magnitude of the cortical effect correlated with the ability to tap along to a beat, suggesting that tracking the beat of complex, ecologically valid music and synchronizing to the beat of simple metronomic stimuli may rely on somewhat overlapping neural resources. Brain imaging studies have shown that both beat perception and beat synchronization are associated with activation in premotor cortex (Grahn and Brett, 2007; Chen et al., 2008; Grahn and McAuley, 2009; Teki et al., 2011; McAuley et al., 2012; Grahn and Rowe, 2013) and increased functional connectivity between auditory and premotor cortex (Chen et al., 2008; Grahn and Rowe, 2009). Individual differences in the strength of the functional connectivity between auditory and motor areas within the cortex could, therefore, be underlying the relationship we find between the effect of beat alignment on stimulus processing and the ability to synchronize to a metronome. Future work could test this hypothesis by correlating the effect of musical rhythm on the neural response to sound and auditory-motor connectivity, either functionally using fMRI or structurally using DTI.

The strength of musical beat perception is difficult to test behaviorally. Tests of tapping to the beat are widely used, but contain a motor production component that cannot be disentangled from beat perception. As a result, the effects of beat alignment on neural responses to sound could be useful for studying rhythm perception in normally developing and impaired populations. It has been suggested, for example, that impaired temporal sampling of slow information is one of the causes underlying reading impairment (Goswami, 2011). Supporting this hypothesis, adults and children with language impairments show greater variability in tapping rates when asked to tap along to a steady beat (Wolff, 2002; Thomson et al., 2006; Thomson and Goswami, 2008; Corriveau and Goswami, 2009), and language-impaired subjects have difficulty distinguishing musical stimuli based on their rhythmic structure (Huss et al., 2011). Our beat alignment paradigm could be used to test the hypothesis that children with dyslexia have impaired processing of musical rhythm. If so, this would lend support to the idea that musical training centered on rhythm could help rehabilitate some language-impaired children (Overy, 2003).

The effect of alignment with the beat on the cortical onset response was found when years of musical experience was included as a covariate, suggesting that stimulus alignment with the beat can affect auditory processing even in populations without specialized training and confirming that this paradigm could be a useful index of musical beat perception in the general population. However, including musical experience as a covariate yielded only a trending effect of alignment with the beat on the brainstem onset response, suggesting that musical experience may play a role in facilitating the modulation of brainstem processing by rhythmic context. This result should be interpreted with caution, given the lack of an interaction and the relatively small number of subjects tested; nonetheless, this finding is consistent with the view that musical experience shapes subcortical processing of sound via corticofugal mechanisms (Kraus and Chandrasekaran, 2010).

One promising avenue for future research would be to investigate whether rhythms presented at different rates have different effects on neural processing. One possibility, for example, is that rhythms within the delta range (1–2 Hz), which is roughly the rate at which stressed syllables tend to be presented during natural speech (Dauer, 1983), have a greater facilitatory effect on auditory processing than do rhythms presented at either a slower or faster rate.

In summary, we found that when a target sound was aligned with the beat of ecologically valid music, as opposed to shifted away from the beat, cortical and brainstem onset responses to the sound were enhanced, demonstrating that the perception of musical rhythm may have robust effects on sound processing in the auditory system. The extent of the cortical enhancement related to the ability to tap to a metronome, suggesting that synchronizing to simple stimuli and tracking the beat of complex stimuli rely on somewhat overlapping neural resources.

### Conflict of interest statement

The authors declare that the research was conducted in the absence of any commercial or financial relationships that could be construed as a potential conflict of interest.
